# Determination of Sudan I and II in Food by High-Performance Liquid Chromatography after Simultaneous Adsorption on Nanosilica

**DOI:** 10.1155/2021/6664463

**Published:** 2021-02-15

**Authors:** Thi Chuyen Pham, Xuan Thu Dang, Bich Ngan Nguyen, Thi Tinh Vu

**Affiliations:** ^1^Faculty of Chemistry, Hanoi National University of Education, Hanoi, Vietnam; ^2^Faculty of Natural Sciences & Technology, Tay Bac University, Son La, Vietnam

## Abstract

Analytical techniques for analyte quantification are often complex, time-consuming, and costly. Further, samples must be carefully prepared to make them suitable for each analytical technique, thus increasing complexity and cost and often requiring toxic solvents. In this paper, we propose a simple and quick method for the pre-concentration of analytes using a nonporous adsorbent: nanosilica, which is prepared from rice husks, an ecofriendly waste material. Subsequently, analysis using high-performance liquid chromatography with a photodiode array detector was used for accurate analyte quantification. To test our method, Sudan I and II dyes were selected because these are potential carcinogens that are often used to adulterate foods because of their bright colors. Although nanosilica has been used as an adsorbent before, the adsorption of hydrophobic organic dyes has not been investigated to date. Thus, the optimal conditions for dye adsorption on nanosilica were systematically studied and found to be 1 mM KCl, pH 3.0, and an adsorption time of 120 min, and the maximum adsorption capacities of the nanosilica for Sudan I and II were 0.619 and 0.699 mg·g^−1^, respectively. The adsorption of the dyes on the nanosilica is discussed in detail with respect to the surface area, functional groups, zeta potential, and adsorption isotherms. Under optimal conditions, the extraction efficiencies of Sudan I and Sudan II reached 98.3% and 92.8%, respectively, and the proposed method was applied for the analysis of several foods and achieved high recoveries (80–100%).

## 1. Introduction

Sudan dyes are azo-type synthetic organic pigments that are widely used industrially because of their fastness and low cost. These substances are classified by the International Agency for Research on Cancer (IARC) as class-III carcinogens. They can cause liver cancer and bowel cancer in animals and increase the risk of chronic human diseases because of their effect on the structure and function of hemoglobin [1, 2]. Therefore, the use of Sudan dyes in food is banned in many countries, and, in some cases, their presence at any level in food is prohibited [3]. Nevertheless, their use continues; in particular, Sudan I and Sudan II are more frequently used in food than Sudan III and Sudan IV because of their eye-catching bright red color. Recently, many analytical methods have been developed to determine the presence of Sudan dyes in food. Among them, LC methods with different detectors are standard. Al Tamim and coworkers used an LC-MS/MS method to simultaneously identify 15 synthetic dyes (including Sudan I and Sudan II) in sauces, cotton candy, and pickle samples [4]. The dyes were characterized by their retention time and multiple reaction monitoring. In their experiments, the limit of detection (LOD) for Sudan I ranged from 2.9 to 3 *µ*g·kg^−1^, whereas the limit of quantification LOQ was found to range from 9.5 to 9.8 *µ*g·kg^−1^. For Sudan II, the LOD and LOQ were 2.8–3.6 *µ*g·kg^−1^ and 10.4–12.8 *µ*g·kg^−1^, respectively. Crucially, the selection of two fragment ion transitions gives this method high sensitivity and precision. In addition, HPLC methods using different types of detectors, such as ultraviolet (UVD) detectors and photodiode array detectors (PDADs), have been widely used for identifying Sudan dyes in food. However, the analysis of Sudan dyes by HPLC with a PDAD detector is difficult because foods are complex matrices, making enrichment difficult and increasing the potential for precipitation in the mobile phase. Therefore, the selection of sample pretreatment procedure is very important.

Currently, there are many modern analytical methods to detect the presence of synthetic pigments in food, for example, plasmonics-enhanced diatomaceous thin films [5], high-performance liquid chromatography (HPLC) [6, 7], liquid chromatography with electrospray ionization-tandem mass spectrometry (LC-ESI-MS/MS) [8], micellar electrokinetic capillary chromatography in combination with ultraviolet (UV) detection [9], reversed-phase (RP) liquid chromatography-tandem mass spectrometry interfaced with electrospray ionization (LC-ESI-MS/MS) [3], and gas chromatography-mass spectrometry [10]. However, although these methods are highly sensitive, they require expensive equipment and are complex, time-consuming, and costly [3, 8, 10]. Further, sample processing is complicated and can require the use of toxic solvents [6, 11–13].

Nanosilica is a well-known adsorbent that has been extensively studied in adsorption science and technology. Nanosilica has been used for the removal of antibiotics [14] and heavy metal ions such as hexavalent chromium [15, 16], surface functionalization for oil adsorption [17], and the fracture toughness enhancement of epoxy resin [18]. Further, nanosilica can be easily fabricated from rice husk and is a low-cost, ecofriendly, and abundant adsorbent [19–22]. Concerning its use as a sorbent, nanosilica has been mainly used to adsorb hydrophilic substances such as methylene blue [23, 24] and Rhodamine B [25], whereas the adsorption of other organic dyes by other adsorbents has been studied intensely [26–31]. However, to date, the adsorption of hydrophobic organic dyes onto nanosilica has not been reported. Crucially, nanosilica has a low point of zero charge (PZC), making it suitable for the adsorption of hydrophobic dyes, yet the adsorption behavior of Sudan dyes by nanosilica and its use for the quantification of these analytes in food have not been studied.

In this paper, we propose a new method to quantify these dyes in food samples at ultra-trace levels. The method is based on the simultaneous pre-concentration and adsorption of Sudan dyes on nanosilica combined with HPLC. The optimum parameters for the adsorption of the Sudan dyes on nanosilica were systematically investigated to determine suitable conditions for Sudan dye enrichment. The proposed method was also applied for the determination of Sudan dyes in different food matrices.

## 2. Experimental

### 2.1. Materials, Instruments, and Apparatus

**Figure 1:**
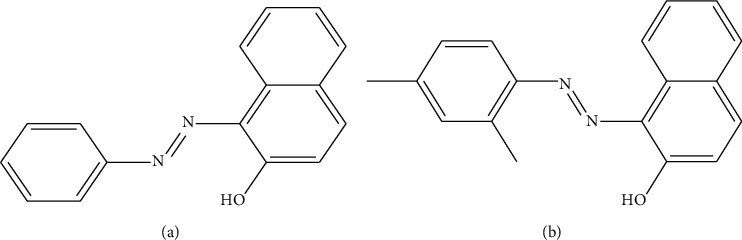
Chemical structures of Sudan I (a) and Sudan II (b).

The rice husk was gifted from the Food Commerce Company (Bac Ninh, Vietnam). Nanosilica was synthesized by the hydrothermal method from the rice husk [14]. Sudan I (1-[(2, 4-dimethylphenyl)azo]-2-naphthalenol) and Sudan II (1-(phenylazo)-2-naphthol) were purchased from Sigma-Aldrich (purity > 99%) and used without further purification. The chemical structures of Sudan I and II are shown in Figure 1. The ionic strength and pH of the sample solutions were adjusted by the addition of KCl (P.A, Merck, Frankfurter, Germany), HCl, or KOH (volumetric analysis grade, Merck). The solution pH was measured using an HI 2215 pH meter (Hanna, Woonsocket, RI, USA). The pH electrode was calibrated with three standard buffers: pH 4.01, 7.01, and 10.01 (Hanna). Organic solvents including ethanol (EtOH), methanol (MeOH), and acetonitrile (ACN) were HPLC grade and purchased from Sigma-Aldrich. Stock solutions (1000 *µ*g mL^−1^) of Sudan I and II were prepared in methanol and stored in dark bottles. The HPLC mobile phases were prepared daily, filtered through a 0.45 *µ*m membrane filter, and then degassed before use. All HPLC analytical solutions were diluted with the mobile phase and filtered through a 0.22 *µ*m membrane filter prior to injection.

Quantification of Sudan I and II was carried out on a Shimadzu HPLC system using an SPD-M20A PDAD. A Waters reversed-phase C18 analytical column (150 mm × 4.6 mm, 100 Å, 5 *µ*m) was used.

### 2.2. HPLC System and Analytical Conditions

Here, the optimized conditions are presented. In Section 3, a discussion of the optimization procedure is given. HPLC measurements were performed with a PDAD detector. The manual injector valve had a 20 *µ*L sample injection volume. A SunFire C18 column (100 Å, 5 *µ*m, 4.6 mm × 150 mm) was used to separate Sudan I and II. The mobile phase comprised methanol, water, and acetonitrile in a 77 : 3 : 20 volume ratio. A constant flow rate of 1.0 mL·min^−1^ was used. The optimum wavelength was 490 nm at a set column temperature of 40°C.

### 2.3. Sample Preparation

To determine the optimal conditions for the extraction of food samples, two dried meat samples, denoted A and B, were used. Fresh pork was purchased from a VinMart supermarket in Hanoi city. Only lean meats were used for the experiments. Lean meat is minced to a maximum size of 5 mm.

First, two equal amounts of ground minced lean meat were placed in two heat-resistant glass cups and 0.1 g NaCl was added. Cup A contained only the meat sample, whereas cup B contained the meat sample and Sudan I and II. A few drops of alcohol were added to allow the infiltration of the dye into the meat and ensure even coloring. Then, the meat samples were stored for one day in a refrigerator to allow the dyes to penetrate the substrate evenly. Then, the samples were mixed well and dried in an oven heated to 50 degrees Celsius. The weight of meat sample B after drying was 10.7717 g in which the weights of Sudan I and Sudan II were 0.0180 g and 0.0231 g, respectively. Dried meat samples were ground and passed through a 0.5 mm sieve. Finally, the sieved samples were further dried in a vacuum oven to a constant mass.

The prepared meat samples were used in the investigation of extraction conditions. To obtain satisfactory extraction efficiency, variables including the extraction solvent, solvent volume, time, and temperature were optimized. It was found that 100% methanol and 100% ethanol had equal extraction efficiency (no significant differences with a 95% confidence probability and *n* = 3), so 100% ethanol was chosen because of its lower toxicity. The optimal extraction parameters are given in Table 1.

The procedure employing adsorption on nanosilica includes the following steps: a weight of 5 g of RHNS was mixed with 50 mL of deionized water and shaken for 2 h to create a homogeneous solution (C). A 10 mL falcon tube consisting of 1 mL of real sample extract, 1 mM KCl, pH 3, 1 mL of solution C, and deionized water was shaken at 30°C for 2 h. Then, the solution was centrifuged 1800 rpm for 30 minutes (10 minutes/time). The desorption of dyes was added by 10 mL of mixed solvent of MeOH, H_2_O, and ACN in a volume ratio of 77 : 3 : 20. The desorption solutions were filtered through a 0.22 *µ*m nylon syringe filter (Xinya Company, Shanghai) before injection onto the HPLC system.

### 2.4. Adsorption Studies

The adsorption capacities (*q*_*e*_) and removal efficiency (*R*_*e*_) of the nanosilica for the Sudan dyes were calculated using equations (1) and (2), respectively [23]:

(1) $$\begin{array}{c} q_{e}=\frac{\left(C_{0}-C_{e}\right).V}{m}, \end{array}$$

(2) $$\begin{array}{c} R_{e}=\frac{C_{0}-C_{t}}{C_{0}}.100\%, \end{array}$$

where *C*_0_, *C*_*t*_,  and *C*_*e*_ are the initial concentrations at time *t* and at equilibrium process (mg·L^−1^), respectively, *V* is the volume of the dye solution (L), and *m* is the mass of the adsorbent (g).

The linearized form of the Langmuir adsorption isotherm equation is given by

(3) $$\begin{array}{c} \frac{c_{e}}{q_{e}}=\frac{c_{e}}{q_{m}}+\frac{1}{\left(q_{m}.K_{L}\right)}, \end{array}$$

where *q*_*m*_ and *K*_*L*_ are the maximum adsorption capacity (mg·g^−1^) and Langmuir adsorption constant of each substance, respectively.

### 2.5. Characterization of the Nanosilica

To study the simultaneous adsorption of Sudan I and Sudan II on nanosilica from rice husk (RHNS), a flask containing 10 mL of adsorbent solution (consisting of nanosilica and Sudan dyes) was shaken in an oscillator at an identical rotational speed at the desired temperature for about 2 h to ensure adsorption equilibrium had been reached. The equilibrium adsorption capacity (*q*_*e*_, mg·g^−1^) was calculated using equation (1). The values of *c*_*e*_/*q*_*e*_ versus *c*_*e*_ were plotted for fitting to the Langmuir adsorption isotherm.

The specific surface area and functional groups of nanosilica after the adsorption of the Sudan dyes were characterized using the Brunauer–Emmett–Teller (BET) method and Fourier transform infrared spectroscopy (FT-IR) [18]. Nitrogen adsorption and desorption isotherms were measured at 77 K using a TriStar 3000 V6.07A (Micromeritics, USA). From the desorption branch of the isotherms, the pore size distribution was calculated using the Barrett–Joyner–Halenda (BJH) method, and the amount of nitrogen adsorbed at a relative pressure of 0.98 enabled the calculation of the pore volume. The FTIR spectra were recorded using an Affinity-1S spectrophotometer (Shimadzu, Japan) at the Department of Inorganic Chemistry, HUS-VNU. All spectra were obtained at a resolution of 4 cm^−1^ and at 25°C and atmospheric pressure.

The change in surface charge after Sudan adsorption was examined using zeta potential measurements. The zeta potential was calculated using the Smoluchowski formula [32] and measurements were carried out using a Malvern Zetasizer (Zetasizer Ver. 7.11, Malvern Instruments, Malvern, UK), and the mean of five replicates was used.

## 3. Results and Discussion

### 3.1. Conditions for the HPLC Analysis of Sudan I and II

**Figure 2:**
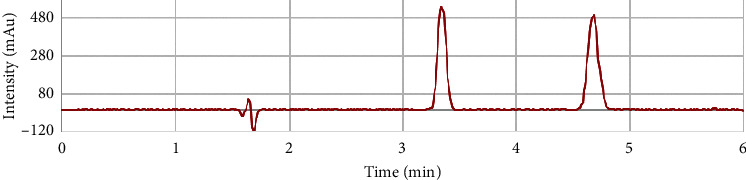
HPLC separation of Sudan dyes I and II.

A mixture containing 1.0 mg·L^−1^ Sudan I and Sudan II standards in ethanol solvent showed a maximum absorption at a wavelength of 490 nm; therefore, we chose this wavelength for further study. We used 5 different types of mobile phases to investigate the appearance of the Sudan dyes and found that a mobile phase of MeOH, H_2_O, and ACN in a volume ratio of 77 : 3 : 20 achieved the best peak shape and separation. The peaks were clearly and completely separated with the following asymmetry coefficients: AS_SudanI_ = 0.963 and AS_Sudan II_ = 0.926. The resolution (RS) was 5.183 (see Figure 2). Repeated measurements showed that the coefficient of variation (CV) was less than 10%. Thus, the data were sufficiently reliable for use to construct a calibration curve. The Sudan I and II standards were added to blank extracts (sample A). The concentration of Sudan I and II was added incrementally from 0.010, 0.020 to 0.100 mg·kg^−1^. The results of statistical processing of the obtained data are in Table 2.

HPLC experiments using the optimized mobile phase at a flow rate of 1.0 mL·min^−1^ and a column temperature of 40°C with the dried meat samples yielded good sensitivity. Even at a very low concentration (0.010 mg·kg^−1^), clear signals with strong absorbance at 490 nm for Sudan I and II were observed. Further, the calculated MQL values were better than those obtained by Iammarino et al. [33], who obtained MQLs of 16 and 22 *µ*g·kg^−1^ with Sudan I and II, respectively, in meat products. In addition, the mean recovery complies with AOAC International standards [34].

### 3.2. Characterization of Nanosilica from Rice Husk

**Figure 3:**
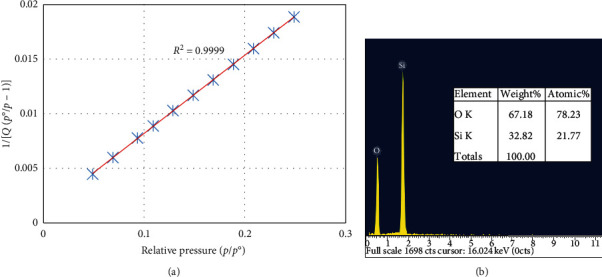
(a) BET isotherms for pore diameter and surface area calculations and (b) EDX spectra showing the presence of Si and O.

The capillary diameter and specific surface area of the nanosilica were determined by the multi-point BET plot method (Figure 3(a)). The results show that the nanosilica material has a capillary diameter (*d*) of 6 nm, making it a medium capillary material (suitable for N_2_ adsorption-desorption). The obtained specific surface area was 57.42 m^2^ g^−1^. Furthermore, the chemical composition of silica was confirmed by energy dispersive x-ray (EDX) spectroscopy measurements, as shown in Figure 3(b), which reveal the presence of Si and O with a total mass percentage of 100%, indicating pure silica.

**Figure 4:**
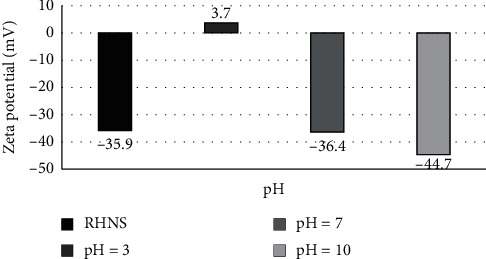
Zeta potential of RHNS with respect to pH.

Figure 4 shows the measured zeta potential with respect to pH for the pristine nanosilica [32] and that after the adsorption of Sudan dyes. A charge inversion occurs when RHNS adsorbs Sudan dyes at pH 3 (from −35.9 to 3.7 mV for pristine to adsorbed, respectively), indicating that, at pH 3, the particles were dispersed in a positively charged suspension and Sudan dye molecules were adsorbed on the surface of the RHNS. Between pH 7 and 10, the zeta potential did not change significantly from that of the pristine nanosilica; thus, at these two pH values, RHNS does not adsorb Sudan dyes significantly, and the negative surface potential changes less because of the negative charge of the base medium.

**Figure 5:**
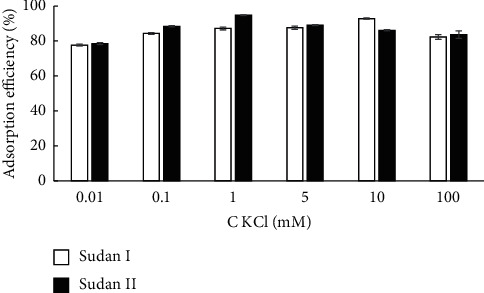
Effect of ionic strength on dye adsorption efficiency (0.01 mM ≥ [KCl] ≥ 100 mM).

To investigate the effect of the ionic strength of the solution on the electrostatic interactions between the nanosilica and Sudan dye, KCl (0.01–100 mM) was added to the adsorbent solution, and this solution was used for adsorption experiments. As shown in Figure 5, when the KCl concentration was increased 100 times from 0.01 mM to 1 mM, the adsorption efficiency for Sudan dyes increased from 78.08% to 90.97%. However, when the KCl concentration also was increased 100 times from 1 mM to 100 mM, the adsorption efficiency for Sudan dyes decreased slightly from 90.97% to 82.97%. The adsorption of dyes decreased slightly because K^+^ partially neutralizes the negative surface charge, which results in a reduction in the attractive forces between the negatively charged adsorbent surface and the dye molecules. At [KCl] < 1 mM, the adsorption efficiency decreased to 72.85% in the absence of KCl; poor adsorption and repeatability were observed. Therefore, 1 mM KCl was selected for further study.

**Figure 6:**
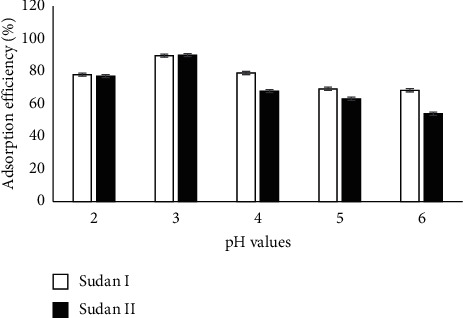
Effect of pH on dye adsorption efficiency at pH 2–6.

The pH of the solution affects the surface charge of the nanosilica and the dissociation the Sudan dyes.  Figure 6 shows the dye adsorption between pH 2 and 6. As shown, the dye adsorption efficiency decreased at pH > 3, indicating that an acidic pH increases the adsorption efficiency.

At high pH values, the surface of the silica is negatively charged [35], and the Sudan azo form is converted to a negatively charged anionic form [36], which reduces the electrostatic attraction between nanosilica and dye significantly. At low pH, the surface of the silica becomes positively charged [35], and the Sudan dyes are neutral. However, the azo group at position 1 (Figure 1) acts as a hydrogen-bond acceptor in the hydrazone form, creating intermolecular hydrogen bonds [36]. In the hydrazone form, the electron density is highly concentrated around the oxygen atom. This increases the van der Waals interactions of the dye with the nanosilica (in the SiOH_2_^+^ form), thereby increasing the adsorption efficiency. At pH 3, the nanosilica and Sudan dyes are almost neutral, so non-electrostatic interactions such as hydrogen interactions or hydrophobic interactions play an important role. In conclusion, pH 3 is optimal for the adsorption of Sudan dyes on the nanosilica.

**Figure 7:**
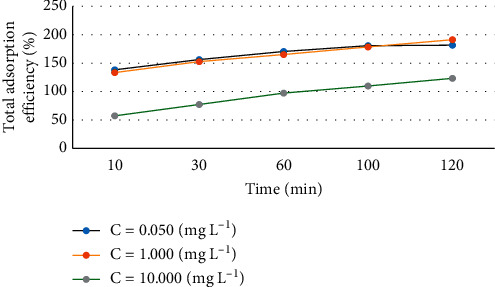
Effect of the initial dye concentration (0.050, 1.000, and 10.000 mg·L^−1^) on the maximum adsorption capacity (*q*_*m*_) with respect to time (10–120 min).

Experiments with initial concentrations of the Sudan dyes of 0.050, 1.000, and 10.000 mg·L^−1^ with adsorption times from 10 to 120 min were carried out. The results in Figure 7 show that, at the three initial concentrations, the optimal adsorption time is 120 min. This implies that, at 120 min, adsorption had reached equilibrium. Thus, 120 min was used for further study. The results also show that when the adsorbent concentration increases too high, effectively the Sudan dyes absorption rate is reduced. Therefore, we choose to investigate the adsorption with the concentration of Sudan dyes less than 1000 *µ*g·L^−1^.

**Figure 8:**
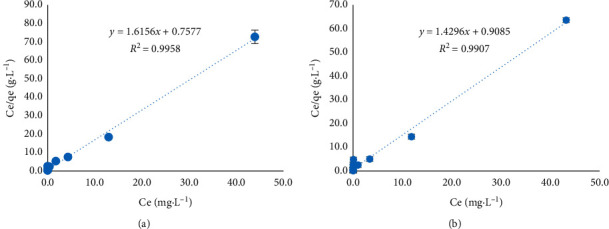
Linear Langmuir plots of Sudan dye adsorption on nanosilica. (a) Sudan I, (b) Sudan II.

Next, Langmuir adsorption isotherms (Figure 8) were plotted to obtain the maximum adsorption capacities. The initial concentrations of Sudan I and II were 30–5000 *µ*g·L^−1^, the adsorption equilibrium time was 120 min, the pH was 3, and [KCl] = 1 mM. The coefficient of determination (*R*^2^) for the linear fit of the data was greater than 0.99, indicating that the dyes are adsorbed by monolayer physical adsorption, and the adsorption centers on the surface are homogeneous [25]. The maximum adsorption capacity (*q*_*m*_) and adsorption constant (*K*_*L*_) for Sudan I were 0.619 mg g^−1^ and 2.132, respectively, whereas those for Sudan II were 0.699 mg g^−1^ and 1.573, respectively.

### 3.3. Analysis of Real Samples

**Figure 9:**
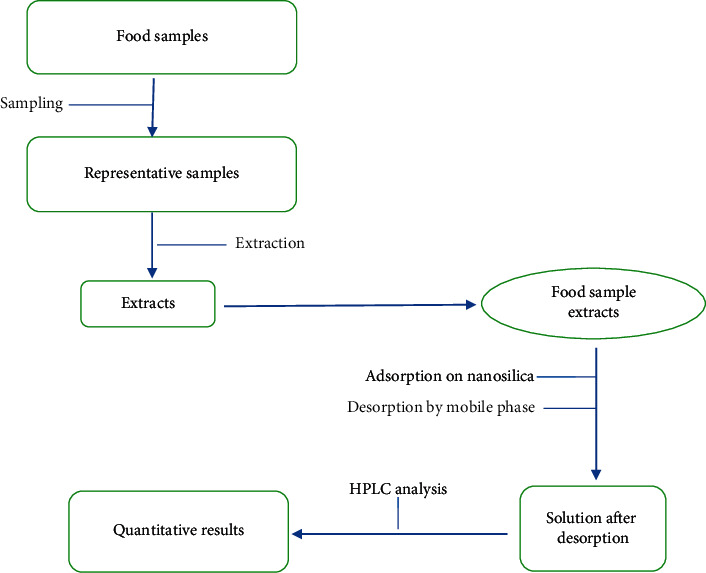
Summary of proposed analytical procedure of food samples.

Samples of dried meats and snacks were purchased from Xanh local market in the Cầu Giấy district of Hanoi, Vietnam. A flow chart showing the procedure for the analysis of the food samples is shown in Figure 9. Because extracts of dried meat samples do not produce suspension when mixed in the mobile phase, it is possible to analyze directly on the HPLC equipment without adsorption by the RHNS. However, extract of snack samples that have precipitation reaction with the mobile phase and direct analysis can damage the chromatographic column. So selective separation of dyes mixture from snacks extract prior to HPLC analysis leads to a solution of practical significance.

Optimal extraction and adsorption conditions were applied to the analysis of three dry meat and five snack samples. The results showed that Sudan I and II were below the LOD in the dried meats samples (1, 3) and snack sample 4. All the remaining samples contained Sudan I and Sudan II (Table 3). To test for matrix effects, two dried meat samples and one snack sample were spiked with Sudan I and II standards at different concentrations. The experiments yielded recoveries of 81.11–100.73% in the real dried meats and 80.74–92.39% in snacks (Table 4). These recovery values are satisfactory according to the AOAC International [34]. This proves that real meat sample matrix has no major impact on accuracy of Sudan dyes quantification.

## 4. Conclusions

In this paper, we have reported the development and optimization of a method for the quantification of a mixture of Sudan I and Sudan II dyes by HPLC. We used a SunFire C18 column (100 Å, 5 *µ*m, 4.6 mm × 150 mm), set column temperature of 40°C, and a MeOH:H_2_O:ACN (77 : 3 : 20 v/v) mobile phase with a constant flow rate of 1.0 ml·min^−1^. The optimal wavelength was 490 nm. The optimal extraction conditions for Sudan dyes from a representative sample (dried meat, 0.02 g) were two cycles of extraction in 10 mL of 100% ethanol at 60°C for 120 min with ultrasonic assistance. Nanosilica made from rice hulls was used as an adsorbent, and the optimal conditions for adsorption were evaluated: 1 mM KCl, initial pH of 3, and adsorption equilibrium time of 120 min. Using the Langmuir isotherm, the maximum adsorption capacity and adsorption constant for Sudan I were found to be 0.619 mg·g^−1^ and 2.132, respectively, and, for Sudan II, 0.699 mg·g^−1^ and 1.573, respectively. The optimum conditions were used to analyze snack samples, revealing that one of the five samples did not contain Sudan I and II, but the remaining four samples contained both Sudan I and Sudan II. Thus, nanosilica from rice husks is suitable for the selective adsorption of Sudan I and II from complex food matrices.

## Figures and Tables

**Table 1:** Optimal sample extraction parameters.

Parameter	Value
Solvent	Ethanol 100%
Solvent volume	10 mL
Ultrasonic vibration	Normal mode
Amount of sample	0.02 grams
Temperature	60°C
Time	120 min
Extraction times	2 times (5 mL solvent/time)

**Table 2:** Method performance and validation parameters.

Chemical	Linear range (mg·L^−1^)	Linear equations	Coefficient	MDL (mg·kg^−1^)	MQL (mg·kg^−1^)	Mean recovery % (*n* = 18)^a^
Sudan I	2 × 10^−3^ to 10	*Y* = (69170.3 ± 590.3) × C	0.9999	0.0022	0.0074	99.83
Sudan II	5 × 10^−3^ to 10	*Y* = (75700.1 ± 371.2) × C	0.9998	0.0029	0.0096	96.16

^a^Three fortification levels (6 repetitions each).

**Table 3:** Quantification of Sudan I and II in food samples (mean ± confidence, *n* = 3).

Sample	Found Sudan I (*µ*g·L^−1^)	Found Sudan II (*µ*g·L^−1^)	Added Sudan I and Sudan II with same concentration (*µ*g·L^−1^)	Recovery of Sudan I (%)	Recovery of Sudan II (%)
Dried meat 1	^*∗*^ND	^*∗*^ND	50,00	82.76 ± 1.13	83.64 ± 0.81
Dried meat 2	26.72 ± 1.48	47.68 ± 1.39	50,00	101.53 ± 1.23	93.24 ± 0.78
Dried meat 3	^*∗*^ND	^*∗*^ND	50,00	84.32 ± 2.25	83.90 ± 2.40
Snack 1	17.59 ± 0.80	44.43 ± 2.57	50,00	77.31 ± 1.06	81.73 ± 2.07
Snack 2	26.85 ± 0.45	40.78 ± 2.20	50,00	82.13 ± 1.03	82.20 ± 1.24
Snack 3	15.97 ± 0.38^a^	28.46 ± 0.43^*∗∗*^	50,00	86.82 ± 0.83	91.22 ± 1.46
Snack 4	^*∗*^ND	^*∗*^ND	50,00	84.98 ± 0.99	86.90 ± 2.31
Snack 5	13.23 ± 0.18^b^	20.39 ± 1.10	50,00	86.49 ± 1.15	86.34 ± 0.99

^*∗*^ND: not detectable. ^a^After desorption, the solution was diluted fivefold with the mobile phase because the contents of Sudan II in spiked snack sample 3 were outside the linear range. ^b^After desorption, the solution was diluted 10-fold with the mobile phase because the contents of Sudan I and Sudan II in spiked snack sample 5 were outside the linear range.

**Table 4:** Recoveries from food samples.

Sample	Spike levels of Sudan I and Sudan II with the same concentration (mg·L^−1^)					
	0.010		0.050		0.100	
	Sudan I	Sudan II	Sudan I	Sudan II	Sudan I	Sudan II
Dried meat 1	82.11 ± 1.45	81.11 ± 1.44	83.44 ± 1.49	85.24 ± 0.70	84.64 ± 1.49	100.73 ± 1.94
Dried meat 3	82.39 ± 1.48	81.78 ± 1.57	84.24 ± 1.93	83.72 ± 2.23	86.52 ± 1.59	84.46 ± 1.69
Snack 4	80.74 ± 1.36	82.40 ± 0.93	81.69 ± 1.42	84.93 ± 2.02	85.15 ± 2.74	92.39 ± 0.95

## Data Availability

The data used to support the findings of this study are included within the article.
